# Malignant hyperthermia and beyond - a virtual BioBank

**DOI:** 10.1186/1471-2253-14-S1-P1

**Published:** 2014-08-18

**Authors:** Rosie Underwood, Nickla Fisher, Dorota Fiszer, Philip Hopkins, Marie-Anne Shaw

**Affiliations:** 1 Leeds Institute of Biomedical & Clinical Sciences, School of Medicine, University of LS9 7TF Leeds, UK; 2 Malignant Hyperthermia Investigation Unit, St James’s University Hospital, LS9 7TF Leeds, UK

## Background

In order to address the complexities of Malignant Hyperthermia (MH) and genetically related disorders it is necessary to access and interrogate easily all information collected over the last 40 years, in addition to incorporating the continuous supply of new information.

## Material and methods

With all types of data held in a single database we are interested in a number of projects including:

• A more detailed analysis of in vitro contracture test (IVCT) data to better define phenotype for research purposes, and to assess the effects of specific mutations on the range of phenotypes. We have previously conducted an analysis of the effects of a number of ryanodine receptor type 1 (*RYR1*) mutations on IVCT and MH reaction phenotypes.

• We have previously published preliminary work considering the possibility of genetic modifiers, and this is a topical area with the role of common versus rare variants as contributors to complex disorders being hotly debated. It will be possible to initiate small projects on the effects of previously identified single nucleotide variants and SNV-SNV interactions on phenotype.

• To analyse observations such as discordancy in greater depth.

• To assess the statistical power for large-scale studies such as a Genome Wide Association Scan.

## Results

In Leeds, we have initiated a relational database, developed using FileMaker Pro®. This software was specifically chosen due to its versatility to set permissions and access rights ensuring that all data stored is kept secure.

A private, restricted access file, currently holds nearly 10,000 records, with one individual being represented as one record. It contains names, family codes, date of birth and diagnostic identification numbers. This file preserves anonymity by assigning individuals within families a unique identification number, subsequently acting to link all other data. This private file is held on a different server from the second wider access ‘public’ file, whose data is linked to the private file only by the unique identifier. This ‘public’ file holds a range of information in the form of tables:

• The family table describes familial relationships, which will be suitable for exporting to .ped files, enabling this data to be imported directly into genetic analysis programs.

• The process table which holds sample collection and storage information, together with the procedures undertaken e.g. histology, exome sequencing, preparation of myoblasts.

• The phenotype and IVCT tables contain information such as clinical details and ‘raw’ IVCT data, allowing for a more detailed analysis in addition to the clinical definition of MHS versus MHN.

• The genotype table contains all genotypic information to date such as mutation testing results.

## Conclusions

We are interested in expanding this database by bringing together Leeds data with other European data. We have the ability to create accounts only for specified users and management through privilege sets, ranging from full access, data entry and read only. This would enable users to assess the feasibility and initiate large-scale collaborative programmes of research. Thus through use of this database, supported by the Leeds centre, we hope to build collectively a pan European resource and international virtual biobank.

